# Cell migration and proliferation capacity of IPEC-J2 cells after short-chain fatty acid exposure

**DOI:** 10.1371/journal.pone.0309742

**Published:** 2024-08-30

**Authors:** Lieselotte Van Bockstal, Sara Prims, Steven Van Cruchten, Miriam Ayuso, Lianqiang Che, Chris Van Ginneken

**Affiliations:** 1 Comparative Perinatal Development, Faculty of Pharmaceutical, Biomedical and Veterinary Sciences, University of Antwerp, Wilrijk, Belgium; 2 Biogenesis Bagó, Development of Biotech Products, Madrid, Spain; 3 Animal Nutrition Institute, Sichuan Agricultural University, Chengdu City, Sichuan Province, China; Southern Illinois University School of Medicine, UNITED STATES OF AMERICA

## Abstract

Novel antimicrobial strategies are necessary to tackle using antibiotics during the suckling and weaning period of piglets, often characterized by *E*. *coli*-induced diarrhea. In the last decades, acetate, propionate, and butyrate, all short-chain fatty acids (SCFAs), have been proposed as an alternative to antibiotics. SCFAs are instrumental in promoting the proliferation of enterocytes, preserving intestinal integrity, and modulating the microbial community by suppressing the growth of pathogenic bacteria in pigs. The effect of individual SCFAs (proprionate, acetate and butyrate) on the regenerative capacity of intestinal cells was investigated via an optimized wound-healing assay in IPEC-J2 cells, a porcine jejunal epithelial cell line. IPEC-J2 cells proved a good model as they express the free fatty acid receptor 2 (FFAR2), an important SCFA receptor with a high affinity for proprionate. Our study demonstrated that propionate (*p* = 0.005) and acetate (*p* = 0.037) were more effective in closing the wound than butyrate (*p* = 0.190). This holds promise in using SCFA’s per os as an alternative to antibiotics.

## Introduction

In pig production, the use of antibiotics peaks during the suckling and weaning period [1], mainly due to the use of hyperprolific sows and the negative effect on the offspring’s resilience combined with the piglets’ relatively young age at weaning. This–often preventive–use of antibiotics contributes to antimicrobial resistance, a critical issue for animal and human health [2]. For this reason, developing novel antimicrobial strategies is crucial [2], and in the last decades, fatty acids have been proposed as a potential alternative [3–8].

SCFAs are the primary products of the gut’s bacterial fermentation of undigested carbohydrates, including acetate, propionate, and butyrate [3, 5, 9]. The most abundant production is seen in the large intestine, where the colonic enterocytes absorb the majority of SCFAs via diffusion or sodium-coupled monocarboxylate transporters. The intracellular actions of SCFAs are mediated via the activation of G-protein coupled receptors, such as free fatty acid receptors (FFARs), and the inhibition of histone deacetylases [3, 10]. FFAR2 was found to be expressed in the small and large intestinal epithelial cells of mice [11, 12]. In addition, butyrate serves as a ligand for transcription factors, such as Foxp3, thereby limiting intestinal inflammatory responses via the production of IL-10 [3, 10]. This way, SCFAs upregulate the expression of tight-junction proteins and increase cell survival. The latter effect is likely related to their ability to enhance the activities of antioxidant enzymes in the enterocytes (e.g., myeloperoxidase and glutathione levels). In addition, butyrate induces the production of antimicrobial peptides [13]. The fraction of SCFAs that is not absorbed lowers the luminal pH and inhibits the potential growth of unwanted microorganisms [3], hence serving as an alternative to antimicrobials [10]. Next to their role in maintaining intestinal integrity, SCFAs are an essential energy source for the enterocytes. The largest body of evidence regarding SCFAs’ antimicrobial effects on the host focuses on butyrate [14–16]. The focus on butyrate might be partly due to its diverse effects and central role in maintaining gut health [10]. The significant disparity in clinical studies on butyrate compared to propionate and acetate, underscores the need for further exploration in this domain [16].

Our hypothesis states that there is a difference in the individual contribution of each SCFA on the wound-healing capacity of IPEC-J2 cells. The perspective is the dietary use of SCFA, which could decrease the intake of antibiotics, by providing energy to the small intestinal epithelial cells, strengthening its barrier function and improving the capacity to repair the intestinal lining, thus reducing the incidence of diarrhea and other intestinal disorders in piglets [2]. In particular, Enterotoxigenic *E*. *coli* (ETEC) represents the largest group of *E*. *coli* causing diarrhea in piglets [17]. Until now it is unknown whether exogenous supplementation of SCFA could increase the resilience of the small intestinal cell lining, by indirectly inhibiting the proliferation of harmful bacteria in the intestinal tract [18]. Literature suggests that SCFAs are equally important in the small intestine as in the colon [19–21].

Intestinal concentrations of SCFAs can be increased via three routes: indirectly by increasing the amount of undigestible dietary fibers, manipulating the microflora composition, or directly by intake of SCFAs [5, 9, 22]. The primary route for suckling piglets to ingest nutrients is via the sow’s milk [23], which is rich in oligosaccharides that can be fermented into SCFAs [24]. A recent study showed an evident influence of the type of milk diet (sow colostrum, bovine colostrum, and milk replacer) on piglets’ gastric, small, and large intestinal microbial communities, resulting in increased SCFA concentrations in the digesta and feces in the case of bovine colostrum [25]. SCFAs–produced by the sow’s gut microflora–can also be ingested by the piglets via the mother’s feces during parturition and post-partum [26, 27]. Interestingly, acetate and butyrate concentrations in the small intestine are similar to those observed in fecal samples (60 mM and 12 mM, respectively). The high activity of the microbes in the large intestine and the small intestine might explain this. In contrast, propionate is 3–5 times lower in small intestine digesta (3 mM) [14, 28], which might be explained by a difference in microbial composition between the small intestine and the colon [28].

This study aims to foster the idea of using SCFAs to support gut health for suckling and weaning piglets. However, given conflicting results on the effects of either orally, rectally, or locally infused SCFAs [29] on *in vivo* small intestinal repair and growth (butyrate [30, 31], propionate [32]), this study aimed to investigate intestinal repair in a standardized *in vitro* set-up: a wound-healing assay using the intestinal porcine cell line IPEC-J2 [33]. These IPEC-J2 cells are neither transformed nor tumorigenic [33]. Albeit recent *in vitro* studies document the beneficial effects of SCFAs using the wound healing assay, these were conducted using Caco-2 cells, which are human colorectal adenocarcinoma cells and mouse small intestinal epithelial cells (MSIE) [34]. Cell lines matching the source epithelium are indispensable for investigating porcine intestinal transport and barrier properties on a subcellular or molecular level and help reduce animal usage [35]. Since we wanted to mimic the effect of individual SCFAs on the porcine intestine, the primary goal of this study was to determine if individual SCFAs could be internalised in the intestinal cells to be able to affect the intestinal epithelial wound healing capacity in IPEC-J2 cells. In this respect, the expression of FFAR2 on these IPEC-J2 cells was verified via western blot and immunohistochemistry. Additionally, the effect on cell proliferation was qualitatively assessed using immunohistochemistry.

Some of the challenges of the IPEC-J2 wound-healing model are the lack of adequate controls and different measuring methods, which limits its comparability and reproducibility [15, 36–40]. Therefore, we optimized the protocol of this wound-healing model to mimic the *in vivo* conditions better and tested epidermal growth factor and spermidine as positive controls. The epidermal growth factor is essential to intestinal cell proliferation [41]. Spermidine, a polyamine produced by the intestinal microflora, was proven to induce epithelial cell proliferation and migration [42, 43]. Hence, another objective of this study was to standardize the wound healing assay in IPEC-J2 cells with a proper negative control and a standard measuring method. This paper aims to answer the following scientific questions: (i) how can we standardize the wound-healing assay in IPEC-J2 cells, (ii) is FFAR2 present in IPEC-J2 cells and pig jejunum samples (iii) and is there a difference between individual SCFA supplementation in the wound-healing capacity of IPEC-J2 cells?

## Materials and methods

### Wound healing assay

**Fig 1:**
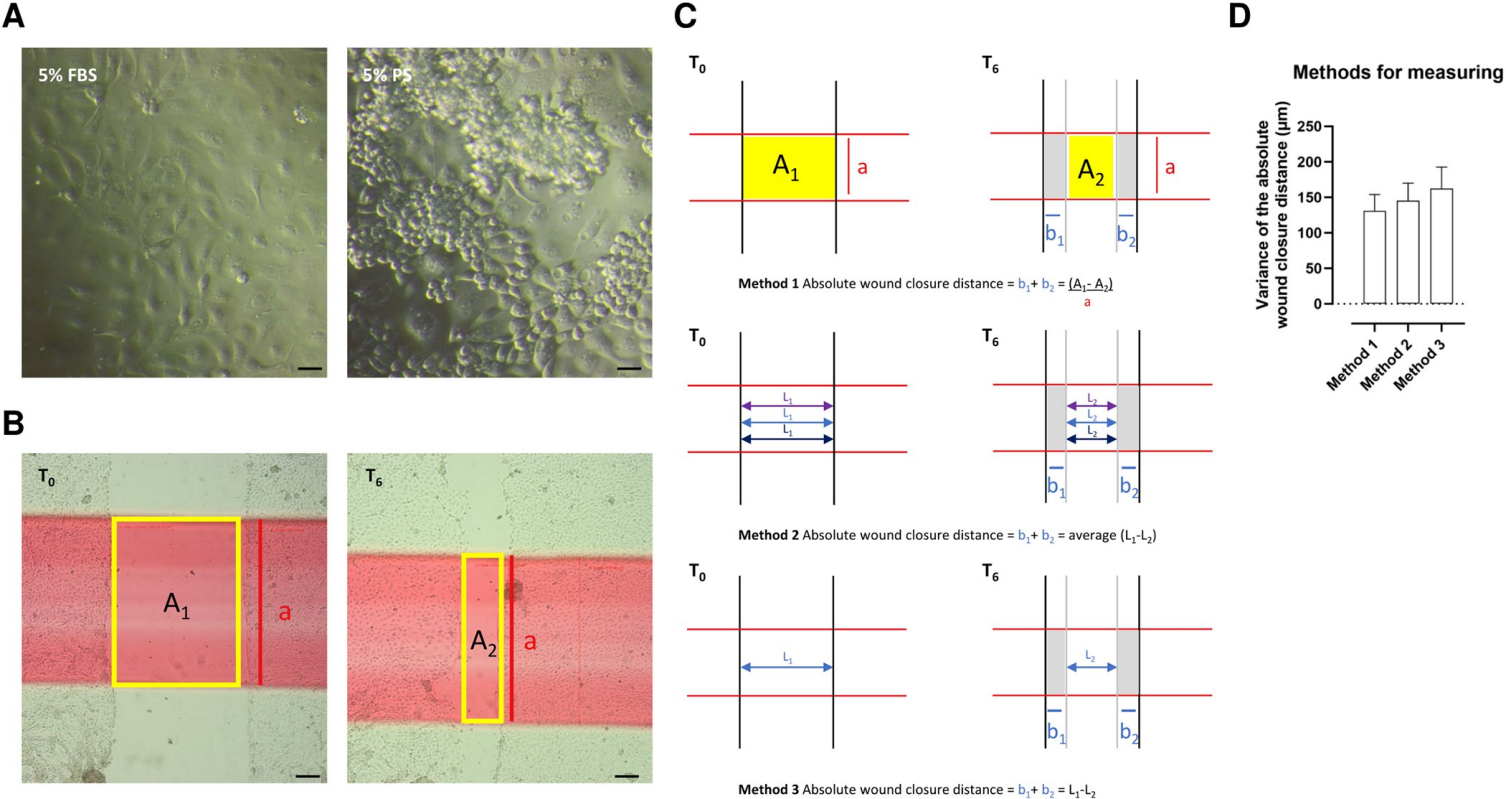
Wound healing assay in IPEC-J2 cells. (**A**) Different growth patterns of IPEC-J2 cells grown in 5% fetal bovine serum (left) or in 5% porcine serum (right) (scale bar = 20 μm). (**B**) Wound healing assay at timepoint 0h when a scratch is made (left) with wound area A_1_ and after 6h (right) with wound area A_2_. The horizontal red line standardises the wound height (a) (scale bar = 200 μm). (**C**) Illustration of the measurement methodologies for calculating the distance to close the wound over 6 hours, which is the sum of both grey distances (b_1_ + b_2_). Method 1 is the methodology used in this paper; method 2 and method 3 are used by other authors. (**D**) Variances (standard deviation) of different methods measuring the absolute wound closure distance. Method 1 is the methodology used in this paper.

Intestinal porcine epithelial cells (IPEC-J2; passage number max 15) (Leibniz Institute DSMZ, Braunschweig, Germany) were seeded in a 12-well plate at a concentration of 100 000 cells/well in growth medium (50% DMEM + 50% Ham’s F12, 5% fetal bovine serum (FBS) or 5% porcine serum (PS), 1% insulin/transferrin/selenium (ITS), 1% penicillin/streptomycin, 1% fungizone and 1.5% HEPES), which was replenished after 24 hours incubation at 37°C and 5% CO_2_. All reagents were purchased at Life Technologies, Darmstadt, Germany. In a first experiment, when a monolayer was obtained, cells were starved for 18 hours with a lean medium 50% DMEM + 50% Ham’s F12 of 1% FBS or 1% PS, 0.5% ITS, 1% penicillin/streptomycin, 1% fungizone and 1.5% HEPES. The goal of the starvation period is to create suboptimal conditions to enhance the effect of the test compounds in experiments two and three. After starvation, a linear scratch was made into the monolayer with a 1,000 μL pipet tip. At that time, the lean medium was replaced with a growth medium (with 5% FBS or 5% PS and 1% ITS). Images were taken at 0h (T_0_) and 6h (T_6_) after scratching, and the area and width of the scratch were measured with Olympus cellSense® imaging software. The following equation calculates the absolute wound closure distance (μm) over 6 hours: [Area T_0_ (μm^2^)—Area T_6_ (μm^2^)]/Standardized height scratch (μm), the latter is height ‘a’ in Fig 1.

In a second experiment, different positive controls were tested: epidermal growth factor (EGF) (Merck, SRP3196) at 5 ng/mL and 50 ng/mL [44] and spermidine (Merck, s0266) at 16 μM were added to the growth medium after starvation of the cells as described for experiment 1 [45].

In a third experiment, the different SCFA’s were tested: sodium acetate (Merck, 1144102) at 0.4 mM, sodium butyrate (Sigma-Aldrich, 303410) at 0.2 mM [46], and sodium propionate (Sigma-Aldrich, P1880) at 0.2 mM were added to the growth medium after starvation of the cells as described for experiment 1. The concentrations for SCFAs were determined based on the concentration ratios *in vivo* [47] and previous viability studies *in vitro* [2, 46]. All experiments were performed in duplicate and repeated three times.

### Western blot FFAR2 and EGFR

Jejunal samples were obtained from humanely killed piglets from another study approved by the Ethical Committee on Animal Experimentation of the University of Antwerp; EC 2015–02. Before removing the jejunum, piglets were killed by terminal anesthesia with sodium pentobarbital (90 mg/kg intraperitoneally) followed by exsanguination. Whole-cell protein was extracted with RIPA buffer from piglet’s jejunum harvested at day 0 and day 28 of age, Caco-2 cells (Leibniz Institute DSMZ, Braunschweig, Germany), and IPEC-J2 cells grown in FBS or PS. Cell lysates were loaded with 2X Laemmli Sample Buffer (Bio-Rad) and heated at 95°C for 10 minutes. Samples and protein ladders (Bio-Rad, 1610374) were then separated by sodium dodecyl sulfate (4–20%) polyacrylamide gel electrophoresis (SDS-PAGE). Gels were run under reducing conditions at 200V for 30 minutes and transferred to nitrocellulose membranes (BioRad). Membranes were blocked for 1h in 5% non-fat dry milk (NFDM, AppliChem) in Tris-buffered saline with tween (TBST; 1X TBS, 0.1% Tween-20). Membranes were then incubated in primary antibodies against FFAR2 (FabGenni, FFAR2-201AP, rabbit polyclonal) at 1:500 dilution, epidermal growth factor receptor (EGFR) (Sigma-Aldrich, E2760, mouse monoclonal) at 1:250 dilution, and β-tubulin (Abcam, ab6046, rabbit polyclonal) at 1:2000 dilution with 5% NFDM in TBST overnight at 4°C. After incubation with the primary antibodies, membranes were incubated with horseradish peroxidase (HRP)-conjugated secondary antibody (Agilent) at 1:2000 dilution for 1h at room temperature. SuperSignal West Pico PLUS Chemiluminescent substrate (Thermo Fischer Scientific, 34579) was used for detection. Membranes were imaged using ChemiDoc XRS+ (BioRad) with Image Lab 5.1 software. The density of the FFAR2-positive bands was normalized to the density of β-tubulin.

### Immunohistochemistry FFAR2 and EGFR

IPEC-J2 and Caco-2 cells were maintained in 50% DMEM + 50% Ham’s F12, 5% fetal bovine serum (FBS) or 5% porcine serum (PS), 1% insulin/transferrin/selenium (ITS), 1% penicillin/streptomycin, 1% fungizone and 1.5% HEPES, respectively in DMEM low glucose medium supplemented with 10% FBS, 1% NEAA, 1% NaP and 0.5% penicillin/streptomycin. Cells were seeded in Nunc chambers (Novolab, A45561) at 1500 cells/well. After a monolayer was grown, cells were fixated with 4% paraformaldehyde for 10 minutes at room temperature. Subsequently, cells were washed with PBS and stored at 4°C until further staining. Cells were incubated for 90 minutes with the primary antibody against EGFR (Sigma-Aldrich, E2760, mouse monoclonal) at 1:20 dilution and FFAR2 (FabGenni, FFAR2-201AP, rabbit polyclonal) at 1:25 dilution at room temperature. Cells were washed with PBS and incubated for 60 minutes with a secondary peroxidase-conjugated antibody against mouse and rabbit (EnVision, Agilent). Subsequently, DAB (Agilent) was added for 5 minutes. Cells were counterstained with hematoxylin for 15 seconds and washed three times.

Intestinal neonatal piglet samples (see above) were fixated in 4% paraformaldehyde, dehydrated by increasing alcohol concentrations, and embedded in paraffin. Next, tissue sections were stained with EGFR and FFAR2 using the protocol described above for the IPEC-J2 and Caco-2 cells. Following staining, samples were washed several times with deionized water and mounted with DPX (Sigma-Aldrich). All samples were imaged using an Olympus CKX41 light microscope.

### Immunohistochemistry cell proliferation

IPEC-J2 cells in porcine serum were grown in 24 well plates and scratched as described in the wound healing assay section. 6 hours after scratching (T_6_), cells were fixated with 4% paraformaldehyde and stored in PBS at 4°C until further staining. Cells were incubated for 90 minutes with a primary antibody against Ki67, a nuclear protein associated with cellular proliferation (DAKO, M7240, mouse monoclonal) at 1:50 dilution at room temperature. Cells were washed with PBS and stained with a peroxidase-conjugated anti-mouse secondary antibody (EnVision, Agilent) for 60 minutes. Subsequently, DAB (Agilent) was added for 5 minutes. Cells were counterstained with hematoxylin for 15 seconds. Following staining, samples were washed several times with deionized water and imaged using a light microscope.

### Statistics

Data analysis was done in GraphPad Prism, considering *p* < 0.05 statistically significant. A Mann-Whitney test was performed for the starvation data, and a one-way ANOVA test with Dunnett’s multiple comparisons test was used for the positive controls and SCFA data. A post-hoc analysis was performed to calculate the power of the study using G*Power analysis. Graphs were made with GraphPad Prism.

## Results and discussion

### Optimalization of the wound healing assay

Most wound healing assays are performed with IPEC-J2 cells cultured in fetal bovine serum (FBS) [48]. However, we opted to use porcine serum (PS), as it corresponds better to the physiological condition of the gastrointestinal tract in piglets. As shown in Fig 1A, the IPEC-J2 cells cultured in 5% FBS formed a monolayer of flattened cells, while IPEC-J2 cells cultured in 5% PS formed a monolayer where more cuboidal cells pile up, seeming to form villi. In addition, the wound closure distance was not markedly different between IPEC-J2/FBS and IPEC-J2/PS (*p* = 0.617), an observation confirming what was reported by Zakrzewski and colleagues [35].

Fig 1B illustrates the wound healing assay at T_0_ and T_6_. At T_6_ the ‘wound’ is smaller than at T_0_. Fig 1C shows the calculation of the absolute wound closure distance. There are different methods to calculate the absolute wound closure distance. Some authors use the relative wound closure area, represented by the percentage of wound closure [45]. This is the area of the initial wound (A1) minus the area of the wound after 6 hours (A2) divided by the initial wound area (A1), times 100 ((A1-A2)/A1)*100). However, this method induces more variation since the relative wound closure area considers the initial wound width, which is subject to variation. By measuring the absolute wound closure distance over a broad distance (wound height, a) (method 1) and not just at one (method 3) or three points (method 2), as suggested in numerous papers [37, 49], variation is reduced. In this paper, we used method 1, where the absolute wound closure distance is divided by the standardized wound height (a) to make comparison possible and generate a measure irrespective of the measured distance. Fig 1D illustrates the variation between the different methods. No significant difference was seen in the variances (*p* = 0.701). Nonetheless, there is a trend for more variation in the absolute wound closure distance when only using one (method 3) or three (method 2) measurements. This aligns with Liang and colleagues [50], who state that at least 100 width measurements are required to estimate the variance correctly.

**Fig 2:**
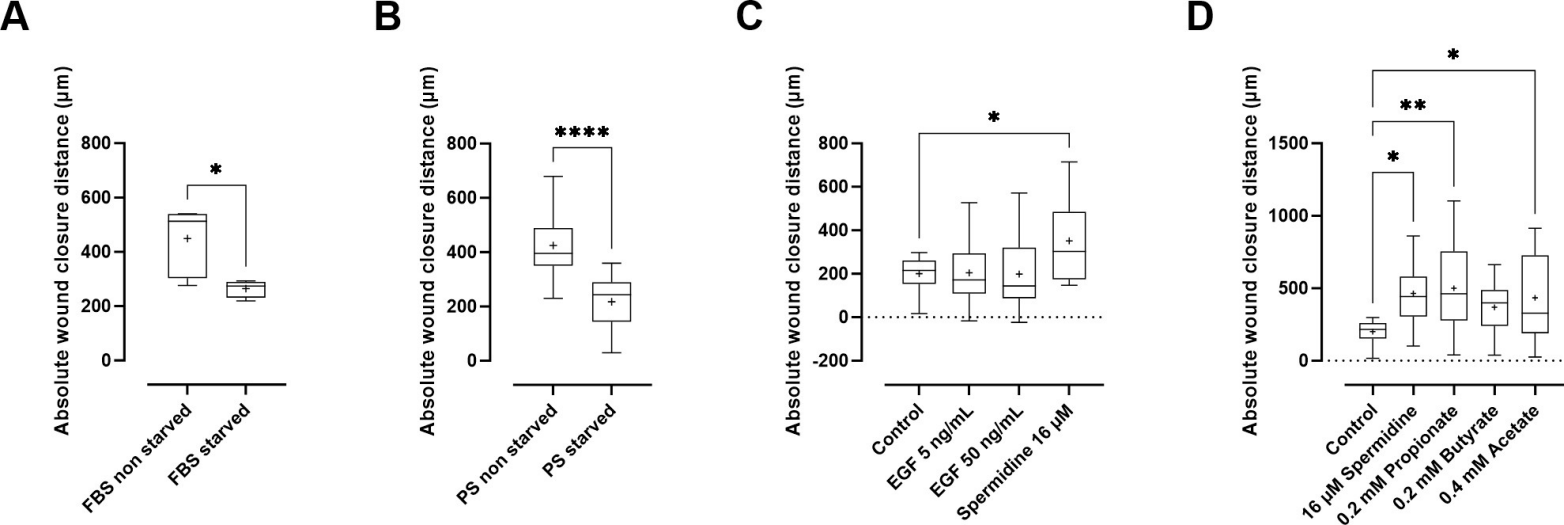
Wound closure distance over 6 hours. (**A-B**) The absolute wound closure distance in different culture conditions. Cells were not starved (5%) or starved (1%) after being exposed to (**A**) bovine (FBS) or (**B**) porcine serum (PS). (**C**) The absolute wound closure distance with different epidermal growth factor (EGF) or spermidine concentrations compared to 1% porcine-starved medium (control). (**D**) The absolute wound closure distance after exposure to different SCFAs and spermidine compared to 1% porcine-starved medium (control). Experiments were performed in three independent repeats run in duplicate. All results are expressed as minimum, first quartile, sample median, third quartile, and maximum. ‘+’ indicates the mean. (* = *p* < 0.05, ** = *p* < 0.01, *** = *p* < 0.001, **** = *p* < 0.0001).

Subsequently, it was hypothesized that the culture medium used was too good to see the effects of the test components. Therefore, a starvation step was added to the protocol since starving cells will stop them from proliferating and migrating [51]. After initiating the wound, a complete medium containing 5% serum was applied to all conditions. Cells stopped growing when adding 0% FBS and 0% ITS (data not shown). Starvation of IPEC-J2 cells in 1% fetal bovine serum (*p* = 0.038; power = 75%) and 1% porcine serum (*p* < 0.0001; power = 99%) significantly reduced the wound closure distance compared to non-starved IPEC-J2 cells in bovine or porcine serum (Fig 2A and 2B). Therefore, starvation of the cells for 18h in a lean medium (1% FBS/PS and 0.5% ITS) was used for the remainder of the experiments.

**Fig 3:**
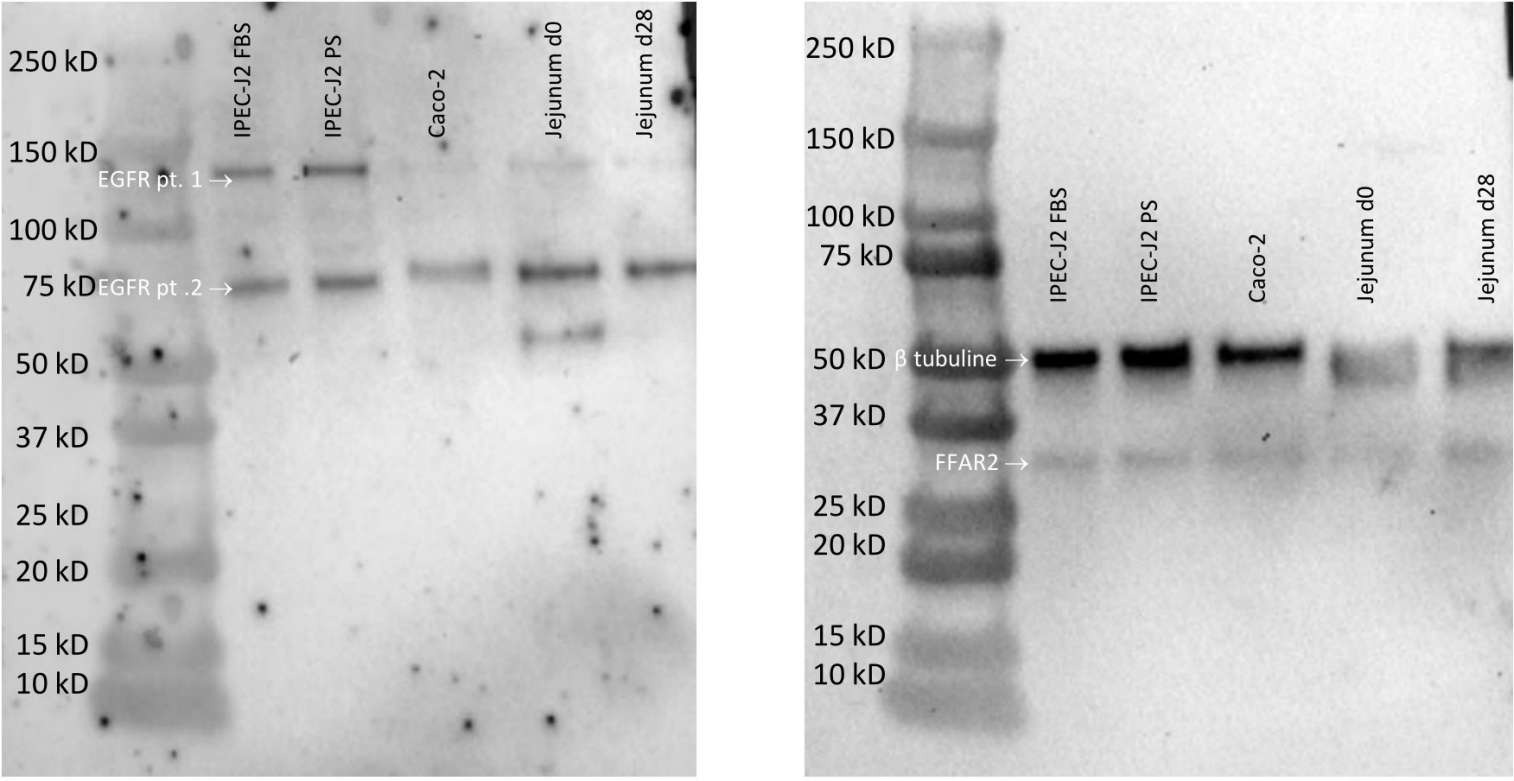
Western blot of EGFR and FFAR2 receptor in different cell lines and intestinal samples. (**Left**) EGFR staining, two bands are visible around 130 kD and 70 kD (**right**), FFAR2 staining (39 kD), and β tubulin staining (52 kD). Samples loaded from left to right: IPEC-J2 cells in 5% bovine serum, IPEC-J2 cells in 5% porcine serum, Caco-2 cells, pig jejunum harvested at day 0 and pig jejunum harvested at day 28.

**Fig 4:**
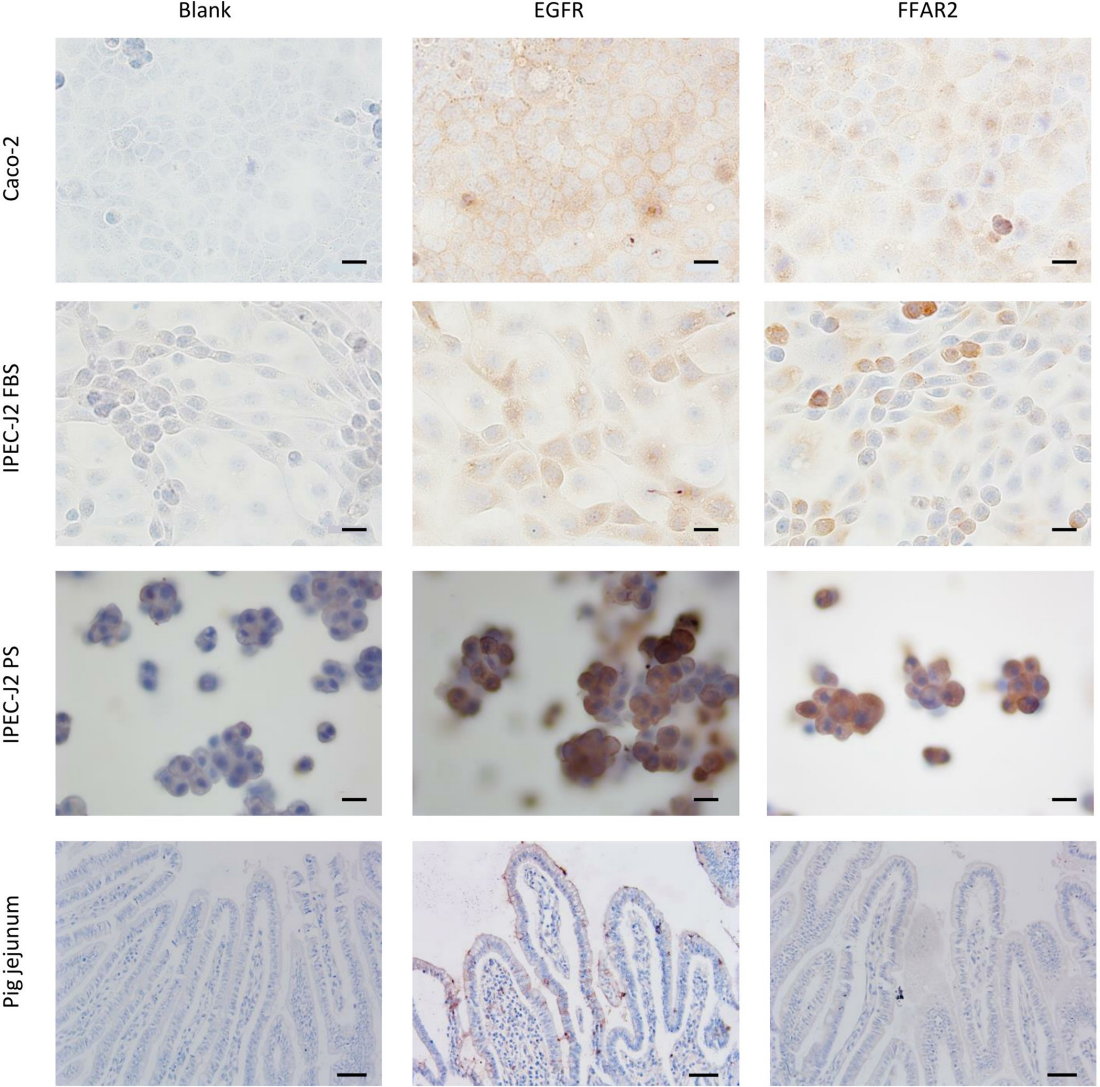
Immunohistochemical staining of EGFR and FFAR2 in different intestinal cell lines and pig jejunum. From left to right: negative control without a primary antibody, EGFR as primary antibody, FFAR2 as primary antibody. From top to bottom: Caco-2 cells, IPEC-J2 cells with 5% bovine serum, IPEC-J2 cells with 5% porcine serum, and below are histological samples of pig jejunum. (scale bar Caco-2 cells and IPEC-J2 cells = 20 μm; scale bar pig jejunum = 50 μm).

Next, a proper positive control was sought (Fig 2C). Although it is reported that EGF could be used as a positive control for wound closing [52], there was no significant difference in the absolute wound closure distance between the negative control and EGF (*p* = 0.999 for EGF at 5 ng/mL; *p* > 0.999 for EGF at 50 ng/mL; power = 86%). A possible explanation is that the wound healing assay is mainly based on cell migration rather than cell proliferation [48], while EGF is primarily responsible for cell proliferation [53]. Since EGF was not found to affect the absolute wound closure distance, we investigated if IPEC-J2 cells express the EGFR. EGFR is expressed in IPEC-J2 cells, Caco-2 cells, and porcine jejunum, as shown with Western blot in Fig 3. We found double bands for EGFR in WB despite using protease inhibitors to avoid protein breakdown [54]. Proteins with post-translational modifications or numerous isoforms can also cause several bands [54]. Indeed, previous studies have shown that isoforms of EGFR exist in human endometrial cancer cells and human breast cancer cells [55–57]. Reiter and colleagues found 60 kDa and 110 kDa isoforms in the human placenta [57]; both molecular weights are similar to our findings. However, this is the first report identifying different isoforms of EGFR in Caco-2 cells, IPEC-J2 cells, and piglet jejunum. The location of EGFR was documented using immunohistochemistry. Fig 4 illustrates the expression of EGFR with immunohistochemistry on Caco-2 cells, IPEC-J2 cells, and pig jejunum tissue. EFGR was visible in the cytoplasm of Caco-2 cells, IPEC-J2 cells, and enterocytes of pig jejunum. Although EGFR is a transmembrane tyrosine receptor, a study by Yu-Ping et al. found a similar cytoplasmatic expression of EGFR in the small intestine [58]. Although we did not see any effect of EGF on the wound closure distance, it was not because of a lack of expression of EGFR on IPEC-J2 cells. As mentioned earlier, EGF’s main mechanism of action is probably cell proliferation rather than cell migration. Alternatively, spermidine could be a positive control in wound healing assays [45]. Spermidine was found to be involved in the early phase of the repair process in wound healing assays, indicating the beneficial role of spermidine in epithelial cell proliferation and migration [42, 43]. Indeed, a significant difference between spermidine and the negative control was found (*p* = 0.015; power = 86%).

### Effect of SCFAs in an IPEC-J2 wound healing assay

To verify whether the individual SCFAs could bind to the IPEC-J2 cells, we investigated the presence of what we consider in this study the most relevant SCFA receptor, FFAR2 (or GPR43) [47]. FFAR2 recognizes acetate, butyrate, and propionate with affinities that differ between species [10]. In general, FFAR2 has a higher affinity for propionate than acetate and butyrate [59], and its expression is restricted to the intestine and immune cells [47]. Its activation modulates inflammation, the secretion of glucagon-like peptide-1, cell spreading and cell polarization [34]. Figs 3 and 4 illustrate the expression of FFAR2 with western blot and immunohistochemistry, respectively, in Caco-2 cells, IPEC-J2 cells, and pig jejunum. No antibody with confirmed cross-reactivity to pigs was commercially available. Therefore, the antibody specificity and protein presence/size (WB) were assessed next to the histological localization of the receptor (IHC). FFAR2 is visible in the cytoplasm of Caco-2 cells, IPEC-J2 cells, and enterocytes of pig jejunum. Although FFAR2 is a transmembrane G-protein-coupled receptor [47], staining of the human colon shows cytoplasmatic expression of the receptor [60]. Although other receptors can bind and facilitate the action of SCFAs, we did not include these in our further analysis. GPR41 (or FFAR3) [59] is more ubiquitously expressed (also in adipose, nervous, and renal tissue), and its activation affects lipid metabolism [61], which is less relevant to our research question. The olfactory receptor 78 is only responsive to acetate and propionate and is expressed in the large intestine, nervous, and vascular tissue. It acts as a hypoxia sensor, which is less critical in our setting [62]. The remaining receptor, GPR109A, only binds with butyrate and is expressed in the intestines and immune cells. It plays a role in suppressing inflammation [10].

The effect of SCFAs on wound healing capacity was tested *in vitro*, including negative (PS-starved cells) and positive (16 μM spermidine) controls. Fig 2D shows that propionate at 0.2 mM (*p* = 0.005; power = 90%) and acetate at 0.2 mM (p = 0.037; power = 90%) significantly increased the absolute wound closure distance compared to the starved control. However, butyrate at 0.2 mM had no effect (*p* = 0.190; power = 90%). Generally, the highest concentration of SCFA in the intestinal lumen is acetate, followed by propionate and butyrate, with a ratio of 60-20-20 [47]. This ratio also applies to our experimental setup, with the highest concentration of acetate (0.4 mM) and lower propionate and butyrate concentrations (0.2 mM). A study by Saleri and colleagues used similar concentrations to investigate the effect of SCFA on intestinal viability and maintenance of intestinal integrity in IPEC-J2 cells. They found that the effect of SCFA depended on the type and specific concentration of the fatty acid. Also, butyrate showed different effects than acetate and propionate [2], which is confirmed by our study. Acetate and propionate sustain cell viability, lower oxidative stress, and maintain intestinal barrier function, while butyrate did not stimulate these parameters [2]. However, an *in vivo* study by Kien and colleagues showed an increase in cell proliferation after cecal butyrate infusion at a physiologically relevant rate [63]. Yan and colleagues found an optimal butyrate concentration in IPEC-J2 cells ranging from 0.1 mM to 1 mM [46], a range compatible with what was used in our study, and showed that concentrations of 2 mM and higher induces apoptosis in Caco-2 cells [64]. Park and colleagues evaluated the small intestinal organoid development in mice, showing an equal increase in organoid area after supplementation of 0.5 mM acetate, 0.5 mM propionate, and 0.5 mM butyrate, but a significantly higher increase when a mixture of these SCFAs was added [65].

While the outcome of our study leads to valuable insights regarding the wound healing capacity of individual short-chain fatty acids on the level of IPEC-J2 cells, there are limitations in its applicability to the *in vivo* situation. Investigating the effect of a combination of acetate, butyrate and propionate mixture as used by Giromini and Park and colleagues [65, 66] on the wound healing capacity of IPEC-J2 cells would be valuable in future research prior to testing *in vivo*. Furthermore, an *in vivo* study where both per oral supplementation of individual SCFA’s and a mixture of these SCFAs are evaluated in physiological concentrations [67] in terms of reducing the incidence of diarrhea in neonatal piglets would be the next step.

### Mucosal repair: Migration vs. proliferation, which mechanism?

A study by Pearce and colleagues investigated the effects of SCFA on the proliferation of human and mouse enterocytes [14]. Butyrate (at concentrations of 5 mM and higher) [68] and propionate caused a complete cessation of cell proliferation [14]. In contrast, Ma and colleagues found that supplementing IPEC-J2 cells with 4 mM sodium butyrate significantly promoted wound healing after 48 hours [15]. Both studies confirm the existence of different repair mechanisms in the intestinal mucosa. Indeed, the repair of intestinal mucosal injury is divided into two independent processes. Firstly, mucosal epithelial cells make up the damaged site through migration, while the cells do not proliferate [45]. Then, the cells supplement the lost cells through proliferation and differentiation [45]. At the concentration used in this experiment, butyrate promotes the differentiation of IPEC-J2 cells. Higher concentrations induce cell apoptosis and promote lysosome formation [69]. To investigate the difference in migration and proliferation capacity of the different compounds, we used the proliferation marker Ki67 [53, 70].

**Fig 5:**
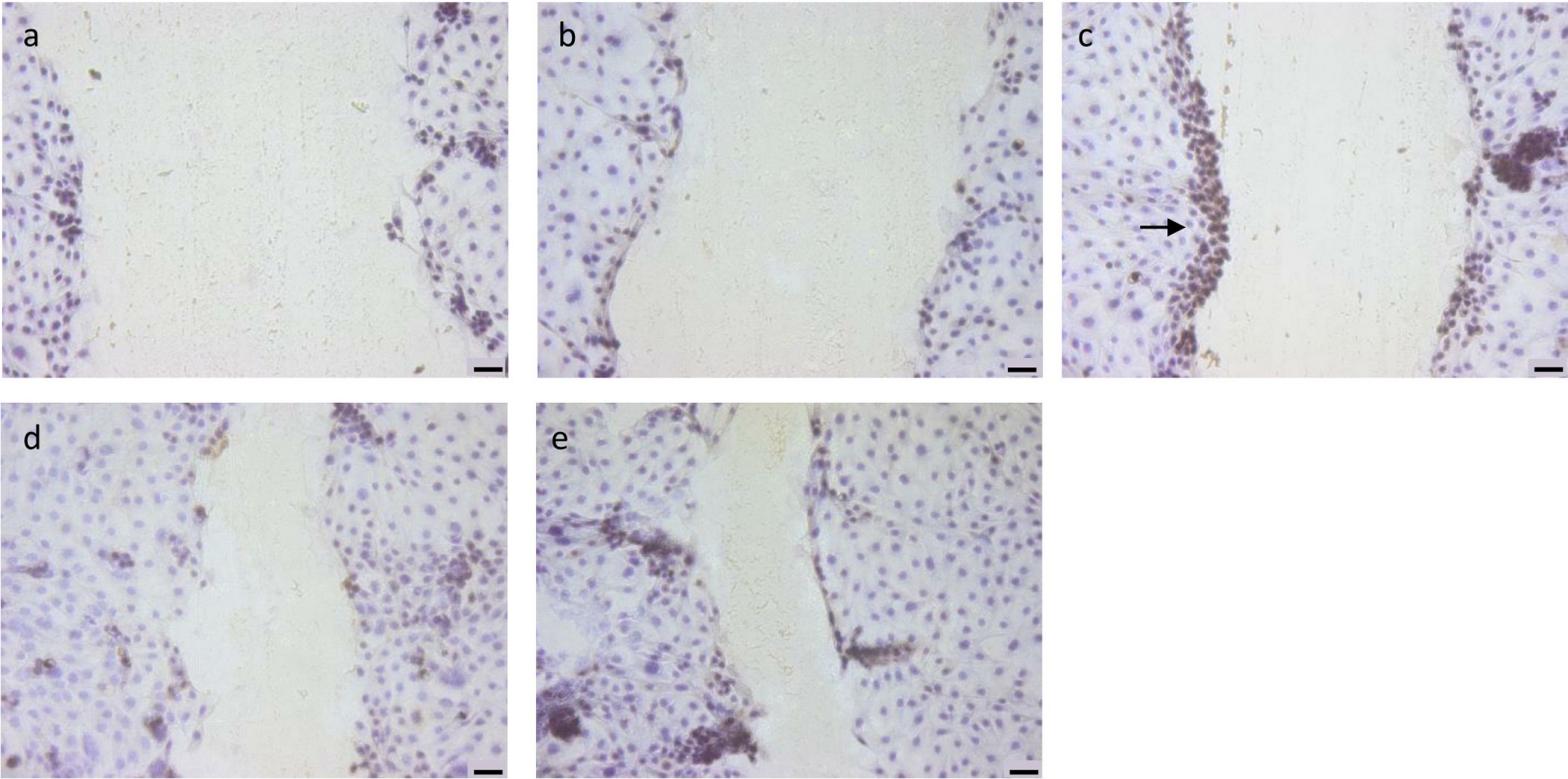
Effect of different growth factors on the proliferation capacity of IPEC-J2 cells in a wound healing assay immunohistochemically stained against Ki67. All images were taken at T_6_. (**a-b**) controls (**a**) negative control without primary antibody (**b**) primary antibody without treatment group (**c-e**) treatment groups (**c**) EGF 5 ng/mL (**d**) spermidine 16 μM (**e**) propionate 0.2 mM. The black arrow shows the proliferation of positive Ki67 cells in the EGF condition. (scale bar = 50 μm).

Previously, it was found that EGF at 100 ng/mL promotes cell growth in IPEC-J2 cells [44]. Another study showed that IPEC-J2 cells supplemented with 5 ng/mL EGF significantly enhanced cell proliferation [71]. This finding contrasts with our study, where supplementation of 5 ng/mL EGF or 50 ng/mL EGF did not markedly affect wound closure. However, Ki67 immunohistochemistry showed a clear difference between cells treated with EGF, spermidine, and propionate (Fig 5). The wound edges are Ki67 positive in the case of EGF treatment (Fig 5C), in contrast to spermidine (Fig 5D) or propionate (Fig 5E) treatment. This phenomenon could be explained by propionate and spermidine improving the repair process through migration, while EGF repairs the damaged site through proliferation, as confirmed by other authors [34, 72].

The current study used a model of porcine intestinal epithelial cells to mimic wound healing after intestinal mucosal damage *in vivo*. We propose incubating IPEC-J2 cells in a lean porcine medium and using spermidine as a negative and positive control respectively. FFAR2, an important receptor for SCFAs, is present in both IPEC-J2 cells and piglet jejunal samples. In this improved wound-healing assay, it was shown that propionate (at 0.2 mM) and acetate (at 0.2 mM) were more effective than butyrate (0.4 mM). Moreover, the repair mechanism of propionate works via the migration of cells rather than the proliferation of enterocytes.

## Supporting information

S1 Raw images Original blot of EGFR and FFAR2 receptor in different cell lines and intestinal samples. (PDF)
