# Supplementary material for: Cell migration and proliferation capacity of IPEC-J2 cells after short-chain fatty acid exposure
Source: PLoS One. 2024 Aug 30;19(8):e0309742. doi: 10.1371/journal.pone.0309742 (PMC11364292; doi:10.1371/journal.pone.0309742)
Supplement: S1 Raw images — (PDF) [file pone.0309742.s001.pdf]

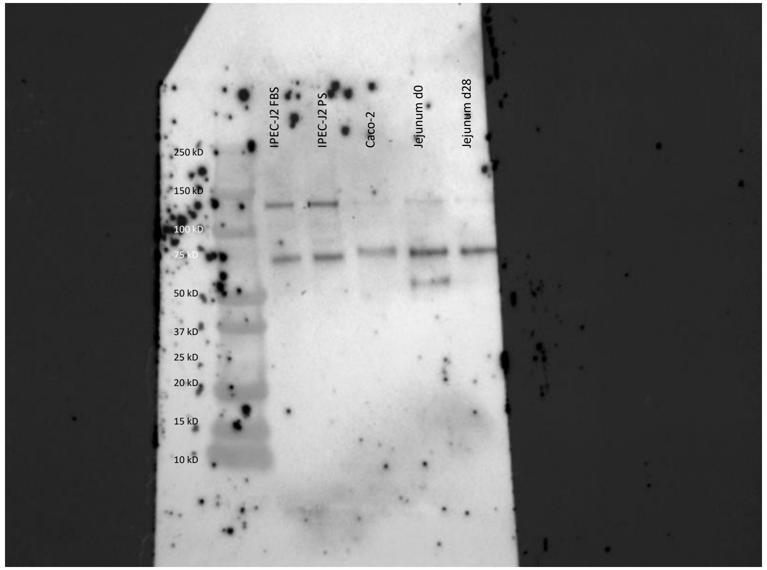

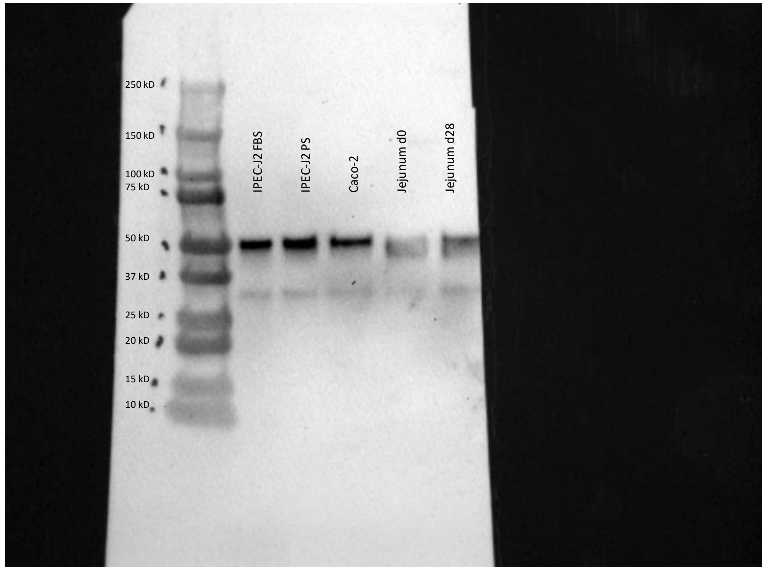

Original blot of EGFR and FFAR2 receptor in different cell lines and intestinal samples. (**Left**) EGFR staining, two bands are visible around 130 kD and 70 kD (**right**) FFAR2 staining (39 kD) and β tubulin staining (52 kD). Samples loaded from left to right: IPEC-J2 cells in 5% bovine serum, IPEC-J2 cells in 5% porcine serum, Caco-2 cells, pig jejunum harvested at day 0 and pig jejunum harvested at day 28.
